# Book Reviews

**Published:** 1918-04

**Authors:** 


### BOOK REVIEWS

Books mentioned may be inspected at and ordered through this office. So far as possible, books received in any month will be reviewed in the issue of the second month following. Pamphlets, quarterly and similar periodicals, reports, transactions, etc., will, as a rule, merely be mentioned.

**Transactions** of the 23rd Annual Meeting of the Am. Laryngological, Rhinological and Otological Society, held in Atlantic City, N. J., May 31-June 1, 1917.

**Transactions** of the American Surgical Association; Vol. XXXV—1917. Dr. John F. Binnie, recorder of the association, edited the volume.

**Military Ophthalmic Surgery.** By Allen Greenwood, M. D., Major, M. R. C., U. S. A., Recently Honorary Lieutenant Colonel, Harvard Surgical Unit, with the Royal Army Medical Corps, British Expeditionary Force. Including a Chapter on Trachoma and Other Contagious Conjunctival Diseases, by G. E. deSchweinitz, M. D., Major, M. R. C., U. S. A., and a Chapter on Ocular Malingering, by Walter R. Parker, M. D., Major, M. R. C., U. S. A. Medical War Manual No. 3. Authorized by the Secretary of War and under the Supervision of the Surgeon General and the Council of National Defense. Illustrated. Philadelphia and New York: Lea & Febiger, 1917. Pp. 115. (Price, $1.50).

This handy volume is intended especially for use in the dressing stations and hospitals of the war zone and presents surgical methods that have stood the test in the British Army hospitals. About ten per cent. of the soldiers in Base Hospitals require some attention to the eyes and surrounding structures. Of the remaining cases, a considerable number will require fundus or visual field examinations at the request of the internist and brain surgeon, and not a few will be found suffering from intra-ocular foreign bodies. As a prophylactic, the author recommends the steel eye shield devised by Dr. W. H. Wilmer for the Ordnance Department. Dr. Greenwood covers concisely the injuries most likely to occur in military practice and gives indications for enucleation and evisceration. The additions by Doctors deSchweinitz and Parker fulfill their purpose admirably. The illustrations, three roentgenograms, five colored plates and 16 cuts, are admirably chosen and well made. It is an excellent manual.

---

**Military Orthopedic Surgery.** Prepared by the Orthopedic Council. Medical War Manual No. 4. Authorized by the Secretary of War and Under the Supervision of the Surgeon General and the Council of National Defense. Illustrated. Philadelphia and New York: Lea & Febiger, 1918. Pp. 240, with supplements of 33 pages. (Price, $1.50).

The Orthopaedic Council, consisting of Majors E. G. Brackett, J. E. Goldthwait, David Silver, F. H. Albee, and Doctors G. G. Davis, A. H. Freiberg, R. W. Lovett and J. L. Porter, in making a manual for military use, do not hesitate to avail themselves of the contributions of our Allies to this most important subject. They have quoted largely from Colonel Sir Robert Jones and Col. Edward L. Munson, U. S. A., and have added a Supplement, profusely illustrated, made up mostly from the "splint book" prepared by the Board of Medical Officers in France. The first 56 pages give excellent directions for the care and treatment of the soldier's foot. The following chapters deal with the joints, the spine, treatment for disabilities following nerve injuries, united and malunited fractures, bone grafting, and methods of fixation. Over 100 illustrations add to the utility of the text.

---

**Food for the Sick.** A manual for Physicians and Patient. By Solomon Strouse, M. D., Associate Attending Physician, The Michael Reese Hospital; Professor of Medicine at the Post-Graduate School, Chicago, and Maude A. Perry, Dietitian at the Michael Reese Hospital, Chicago. 12mo of 270 pages. Philadelphia and London: W. B. Saunders Company, 1917. Cloth, $1.50 net.

In prescribing diets for his patients there are many things that the doctor does not explain and many ideas that could be amplified with profit to both parties. These things the authors have expressed briefly in their book, giving us not merely 283 diets and 124 special recipes with indications for their use, but prefacing the study of each disease with a discussion of its peculiarities from the view point of the dietitian, and showing the why and wherefore of the dietetic restrictions imposed on the patient. The large number of interchangeable menus under one heading, such as diabetes mellitus, affords the greatest possible variety consistent with the demands of treatment. This makes the book especially useful in suggesting ''something different'' to the mind of the perplexed nurse and physician.

---

**Surgery and Diseases of Mouth and Jaws.** By Vilray Papin Blair, Major M. O. R. C., A. M., M. D., Clinical Professor of Surgery, Professor of Oral Surgery, and late Associate Professor of Anatomy, Washington University, St. Louis. 764 pp., 460 illustrations. Third edition, revised under direction of Surgeon-General U. S. Army. Required text for Army Schools and Plastic Surgery. St. Louis, C. V. Mosby Company, 1917. Cloth, $6.00.

The third edition of this comprehensive treatise appears about five years after the first. There are a number of additions relating to injury and sepsis and their treatment as modified by the extensive experiences of the war. The subjects of peridental infections, local and general anesthesia have been re-written. There is a generous bibliography and an ample index. Altogether this volume is of the greatest service to the specialist who deals with any of the structures of the mouth and jaws.

---

**Medical Ophthalmology.** By Arnold Knapp, M. D., Professor of Ophthalmology, Columbia University, Executive Surgeon Herman Knapp Memorial Eye Hospital. Cloth, 8vo, 509 pages, 32 illustrations, price, $4.00 net. Philadelphia, P. Blakiston's Son & Co., 1918.

This book is part of the International System of Ophthalmic Practice edited by Walter L. Pyle, A. M., M. D., of Philadelphia. It aims to give the essential relations of ophthalmology with other branches of medicine and surgery. The first eighty pages give an illustrated description of ophthalmological anatomy and physiology with special reference to the anatomy of those parts of the nervous system connected with sight. The rest of the volume takes up in order the various medical and surgical diseases and infections, poisons, and hereditary eye diseases. The range of the work is broad and it will be found very useful as a key to the influence of extra-ocular conditions of the eye itself.

**State Board Examination Questions and Answers of the United States and Canada.** A Practical Work Giving Authentic Questions and Authoritative Answers in Full That Will Prove Helpful in Passing State Board Examinations. Reprinted from the Medical Record. Fifth Edition. Altogether New Matter. Every Question Answered in Full. Price, $2.50. New York: William Wood & Company, 1918.

This octavo of 970 pages presents enough questions from State examinations to acquaint the student with the general character of the papers that doctors are required to pass before obtaining the right to practice medicine. The answers appear to be carefully prepared, with no attempt to economize space. One who studies this book diligently before undertaking a written examination ought to have a fair idea of the nature and scope of the task awaiting him.

---

**A Practical Text-Book of Infection, Immunity and Specific Therapy** with special reference to immunologic technic. By John A. Kolmer, M. D., Dr. P. H., M. Sc., Assistant Professor of Experimental Pathology, University of Pennsylvania, with an introduction by Allen J. Smith, M. D,. Professor of Pathology, University of Pennsylvania. Second Edition Thoroughly Revised. Octavo of 978 pages with 147 original illustrations, 46 in colors. Philadelphia and London: W. B. Saunders Company, 1917. Cloth, $7.00 net, Half Moroco, $8.50.

The appearance of the second edition of this text-book within two years from the first impress is sufficient evidence of its value. The author and the illustrator, Erwin F. Faber, have taken an extremely technical branch of science and treated it in a clear and readable manner. The work is valuable not alone for instruction but for reference and as a hand-book of technic. Part I takes up the general technic of immunology; Part II presents the principles of infection; Part III is a comprehensive discussion of the principles of immunity and the technic of special immunology; Part IV deals thoroughly with applied immunity; Part V is a laboratory guide in experimental infection and immunity, comprising 113 experiments, with suggestive questions for discussion. The revision includes new matter on focal infections, the Schick test, complement-fixation in tuberculosis and other infections, a quantitative Wassermann reaction, studies of the toxicity of salvarsan, new matter on anaphylaxis, chemotherapy, and many other topics, bringing the work strictly up to date.

